# Emerging ctDNA detection strategies in clinical cancer theranostics

**DOI:** 10.1002/SMMD.20230031

**Published:** 2023-11-13

**Authors:** Kexin Yi, Xiaoju Wang, Sergey K. Filippov, Hongbo Zhang

**Affiliations:** ^1^ Pharmaceutical Sciences Laboratory Åbo Akademi University Turku Finland; ^2^ DWI‐Leibniz Institute for Interactive Materials e. V. Aachen Germany; ^3^ School of Pharmacy University of Reading Reading UK; ^4^ Turku Bioscience Centre University of Turku and Åbo Akademi University Turku Finland

**Keywords:** biosensor, cancer theranostics, circulating tumor DNA, digital PCR, next generation sequencing

## Abstract

Circulating tumor DNA (ctDNA) is naked DNA molecules shed from the tumor cells into the peripheral blood circulation. They contain tumor‐specific gene mutations and other valuable information. ctDNA is considered to be one of the most significant analytes in liquid biopsies. Over the past decades, numerous researchers have developed various detection strategies to perform quantitative or qualitative ctDNA analysis, including PCR‐based detection and sequencing‐based detection. More and more studies have illustrated the great value of ctDNA as a biomarker in the diagnosis, prognosis and heterogeneity of tumor. In this review, we first outlined the development of digital PCR (dPCR)‐based and next generation sequencing (NGS)‐based ctDNA detection systems. Besides, we presented the introduction of the emerging ctDNA analysis strategies based on various biosensors, such as electrochemical biosensors, fluorescent biosensors, surface plasmon resonance and Raman spectroscopy, as well as their applications in the field of biomedicine. Finally, we summarized the essentials of the preceding discussions, and the existing challenges and prospects for the future are also involved.

## Highlights

- Outline the development of ctDNA detection strategies based on digital PCR (dPCR)‐based and next generation sequencing (NGS).
- Emerging ctDNA analysis systems based on various biosensors are emphatically introduced.
- Summarize the challenges and the future perspectives for ctDNA detection.

## Graphical abstract

Overview of ctDNA detection strategies in clinical cancer theranostics. In this review, the recent development of ctDNA detection systems based on digital PCR (dPCR), next generation sequencing (NGS) and various biosensors was introduced. The essentials of the preceding discussions are summarized, and the existing challenges and prospects for the future are also involved.

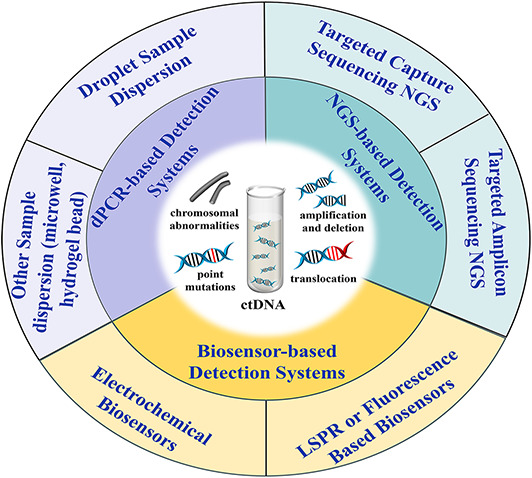

## 1 INTRODUCTION

The tumor tissue refers to the new tissue formed by abnormal proliferation of certain tissue cells under the effect of various tumorigenic factors.^1^, ^2^ Tumors are classified into benign and malignant tumors according to their differentiation degree, growth rate, and other characteristics, and malignant tumors are generally known as cancer. Cancer is a major public health issue all over the world.^3^, ^4^, ^5^ Early detection of malignant tumor tissue and heterogeneity have an impact in decreasing its associated mortality and could even reduce the incidence. Besides, regular repeated detection is of great assistance to monitor cancer progression and treatment response. Conventionally, cancer detection is performed by means including tissue biopsy, ultrasound imaging technology, molybdenum target imaging, and nuclear magnetic resonance imaging and so on.^6^, ^7^, ^8^ Among these, tissue biopsy is considered as a gold standard in the cancer diagnosis. It refers to the removal of pathological tissue from patients' bodies for pathological examination using methods including cutting, forceps, or puncture, as needed for the therapy. Nevertheless, some limitations and defects still exist. These approaches are not informative enough for the early diagnosis and heterogeneity identity of cancer.^9^ In addition, pathological sections for tissue biopsy are obtained by puncturing; this invasive surgical procedure brings patients a great degree of complications, tumor metastasis and injury‐related risks.^10^ Moreover, this also significantly increases the difficulty of performing repeat biopsies. In recent decades, benefiting from the rapid development of molecular diagnostics, the liquid biopsy technology has acquired many remarkable achievements.^11^, ^12^, ^13^ It has been recognized as a promising diagnostic tool for the various cancers.

Liquid biopsy refers to obtain information about the cancer by analyzing various tumor components, including circulating tumor cells (CTCs), exosomes, circulating tumor DNA (ctDNA) and cell‐free RNA (cfRNA).^14^, ^15^ Compared with traditional tissue biopsies, the sampling operation is minimally invasive and convenient for repetition. Real‐time monitoring of cancer progression could be realized by analyzing serial samples. Among multiple available samples, ctDNA has become more and more attractive to researchers due to its excellent superiority. Normally, necrotic or apoptotic cells passively release cell‐free DNA (cfDNA) into plasma. CtDNA specifically refers to the cfDNA secreted by tumor cells; it could be derived from primary tumors, CTCs, micrometastases, or overt metastases, and then released into patients' blood circulation. Its essence is a fragment of tumor cell DNA. As a fragment of the genetic substance of cancer tissue cells, ctDNA contains cancer‐specific genetic aberrations.^16^, ^17^ Additionally, in the heterogeneity perspective, ctDNA could offer a more comprehensive overview of the mutation spectrum present in patients' tumor. Therefore, it is of significant clinical implications to comprehensively and accurately analyze ctDNA properties, such as the size, integrity, known variants, other genetic perturbations and so on. These would be of great assistance in early cancer diagnosis, recurrence detection and prognosis determination, treatment response prediction, real‐time monitoring of the disease courses and assessment of postoperative residual disease.^18^, ^19^ The application range of ctDNA detection in the biomedicine field is also getting further wide.

**FIGURE 1:**
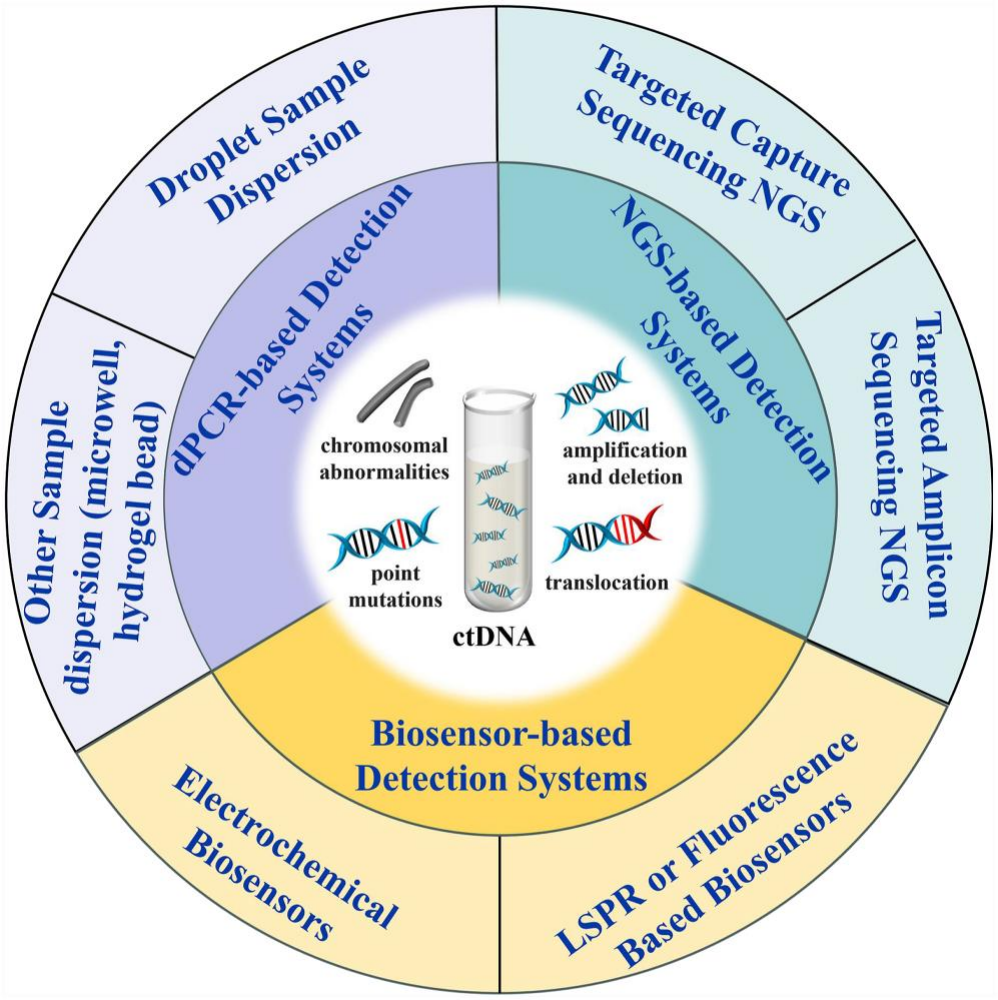
Overview of ctDNA detection strategies in clinical cancer theranostics.

The initial determination methods, such as end‐point PCR, only allowed to acquire the total level of cfDNA. It is difficult to be widely utilized due to the low precision.^20^ With the in‐depth study of ctNDA, it was demonstrated that it could be enriched by a length selection method and blood was the ideal analyte.^20^ In the meantime, PCR‐based ctDNA analysis technologies have also made great progress. They evolved from the PCR‐based target enrichment strategy to the oligomer‐specific hybridization and the allele‐specific quantitative PCR (qPCR). But the high limit of detection (LoD) often reduces their applicability, and it is still difficult to detect rare variants.^21^ The emergence of digital PCR (dPCR) broke these limitations. Nevertheless, a non‐ignorable disadvantage of PCR‐based assays is that the interested potential mutation has to be known in advance. Next generation sequencing provides a method to screen mutations in a broader target space.^22^, ^23^ But the clinical application of single‐locus NGS was relatively limited due to its low recovery rate and narrow genomic space in a single reaction pool. ctDNA detection platforms based on various biosensors provide a countermeasure to address these issues. The efficient and high‐throughput ctDNA analysis, even on‐site detection, is expected to be achieved thanks to their unique superiority of satisfactory specificity, sensitivity, portability and cost‐effectivity.^24^ These rapid developments of various analysis strategies make ctDNA detection more comprehensive and accurate. It could be studied by physical properties analysis and content quantification. Herein, we first reviewed the development of several typical dPCR‐based ctDNA detection methods and NGS‐based ctDNA assay systems. Furthermore, the emerging ctDNA analysis strategies based on different types of biosensors including electrochemical biosensors, fluorescent biosensors, surface plasmon resonance (SPR) as well as Raman spectroscopy are emphatically introduced. Finally, the summary of the above discussion, the remaining issues and future prognostications are also presented (Figure 1).

## 2 dPCR‐BASED ctDNA DETECTION STRATEGIES

### 2.1 dPCR assay systems based on droplet sample dispersion method

PCR‐based assay systems are currently widely applied for the detection of ctDNA.^25^, ^26^, ^27^, ^28^, ^29^, ^30^, ^31^ PCR is a technique in which target DNA molecules are amplified using synthetic deoxyribonucleotide triphosphate (dNTPs), specially designed DNA primers and DNA polymerase. After a certain number of reaction cycles, even a small number of the target DNA molecules could be rapidly amplified to the concentration which is applicative for quantitative assay, and subsequently could be analyzed through fluorescence detection or other methods. In conventional PCR, the target number that could be concurrently quantitated is restricted by the fluorophore number. Besides, there are still some inevitable false amplification of wild‐type sequences due to the randomness of molecular interaction. Although designs of molecular primers and blockers were optimized, it is still challenging to reliably detect low allele frequency variants.

Over recent decades, many strategies to overcome these problems have emerged in the continuous evolution of PCR‐based analysis systems. qPCR has become the broadly accepted relative quantification assay method for DNA detection. The quantification method in qPCR was changed to monitoring all amplification cycles on the basis of the calibration curve. However, its unsatisfactory accuracy was still a limitation. The dPCR technology emerged with its accurate and highly efficient gene analysis function, which possessed the great potential to break this restriction. dPCR is considered to be the 3rd generation PCR technology that consists of 3 major steps: split, amplification, and fluorescence detection.^32^, ^33^, ^34^ During the split process, numerous micro‐or nano‐chambers were created based on the microfluidic technique or the droplet emulsion method. Then, the target molecule in the reaction chamber would be exponentially amplified during the amplification, and generate bright fluorescence in the meantime. These chambers would be marked as positive, while the chambers which emit weak fluorescence (without target molecules) would be marked as negative. Finally, the fluorescence signals were quantitatively analyzed by droplet‐based flow cytometry or optical photo analysis. Compared with qPCR or other conventional PCR methods, dPCR‐based detection systems are provided with distinctive merits such as absolute quantification, extraordinary accuracy and more superior anti‐interference performance.^35^, ^36^ dPCR has been considered as a promising tool for the identification of rare DNA sequence mutants. Therefore, it has been broadly employed in academic and clinical research, including the theranostics of cancers, infectious diseases and other rare diseases.

**FIGURE 2:**
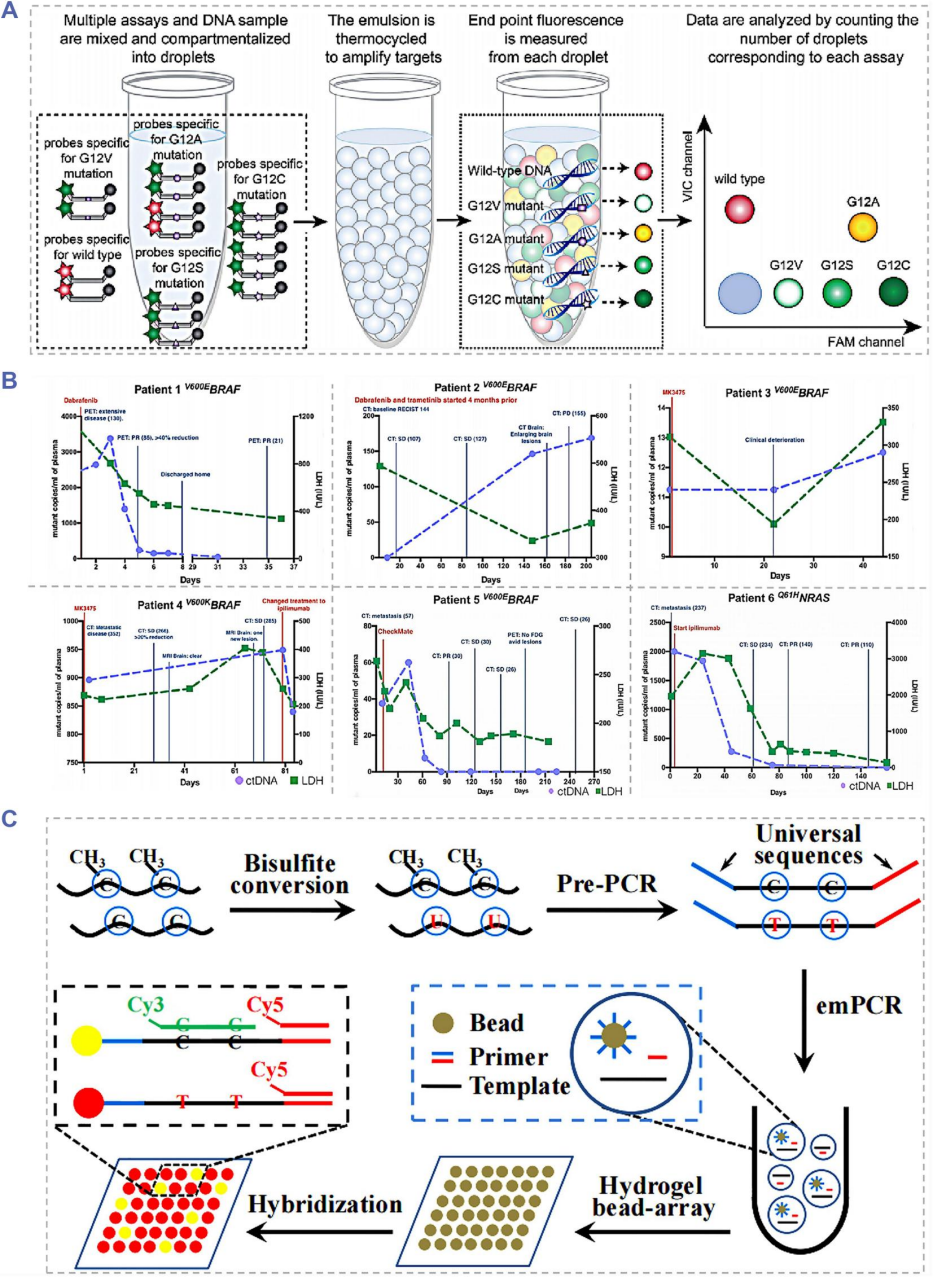
Examples of dPCR‐based ctDNA detection systems. (A) Schematic of the droplet‐based multiplex dPCR for detecting the KRAS mutation in ctDNA of metastatic CRC patients. Reproduced with permission.^37^ Copyright 2013, The American Association for Clinical Chemistry. (B) ctDNA and monitoring with clinical follow‐up for 6 melanoma patients. Reproduced under terms of the CC‐BY license.^42^ Copyright 2015, The Authors, published by Springer Nature. (C) Schematic of the gene methylation assay method combining the emPCR and the hydrogel bead array. Reproduced with permission.^43^ Copyright 2017, Elsevier.

As the earliest dPCR method, droplet‐based dPCR approaches were especially appropriate to quantify the minority nucleic acid biomarker in ctDNA including rare subclones and have made significant achievements in related scientific fields.^37^ Many reports have demonstrated the successful applications of dPCR in the clinical follow‐up of various cancers by analyzing peripheral blood samples.^38^, ^39^, ^40^, ^41^ By applying the droplet‐based multiplex dPCR method, as shown in Figure 2A, Taly et al have successfully realized the detection of KRAS mutation in ctDNA of metastatic CRC patients.^37^ In this study, 50 clinical samples were involved to be assayed by a two‐panel multiplex PCR procedure which could detect the wild‐type sequences and the 7 most frequent mutations in the KRAS oncogene. Moreover, a droplet‐based dPCR system has also been employed in the research of detecting mutated alleles in the blood of melanoma blood.^42^, ^44^, ^45^, ^46^ Chang‐Hao Tsao et al determined the ctDNA levels in samples of patients with stage IV melanoma via droplet‐based dPCR^42^ (Figure 2B). In this work, 6 melanoma patients presented with either a BRAF V600 E, V600 K or a NRAS Q61H mutation (detected by tumor biopsy) were given different treatments, and the ctDNA levels (the copy number of mutated alleles per mL of sample) of their plasma were further applied to monitor the therapy response. The result indicated that the ctDNA level may be more instructive than the conventionally used lactate dehydrogenase marker, and more conducive for the tracking cancer progress.

Overall, the droplet‐based dPCR is able to accurately quantify the target DNA in a relatively simple manner. This system provides the combination of the basic methods of PCR and the microfluidic methods to generate and control droplets, ensuring their uniformity and allowing for their mixing, transfer, and analysis. In addition, the heat transfer rate is significantly increased because of the considerable specific surface area of the microscale droplet system, and the PCR reaction process is thus accelerated. Notably, in the droplet‐based dPCR, the potential cross contamination and non‐specific amplification between droplets could be avoided due to the generation of droplets in the oil phase. However, erroneous results may still occur sometimes due to the possibility of the droplet rupture during the process of transferring and thermal cycling. Therefore, the future development trend of droplet‐based dPCR detection platforms should be the investigation of more sophisticated and multifunctional device design, and the manufacturing method of integration system.

### 2.2 dPCR assay systems based on other sample dispersion method

dPCR assay based on the microwell‐based sample dispersion combines the parallel microarrays and the high specificity and sensitivity as well as the superior quantitative ability of dPCR method.^47^, ^48^ It has become a feasible alternative for water‐in‐oil strategies. The main step of microwell‐based dPCR is to manufacture the microwell array chip, which is constituted of through holes in most cases. At the beginning, the dense micro‐scaled well arrays were constructed on rigid chips, such as silicon chips. Getting benefit from the rapid development of microfabrication technology, microwell array chips or their surfaces are modified or through physical or chemical methods to become hydrophobic. Consequently, these microwells become nanoscale microreactors where thousands of single molecule amplification reactions could occur simultaneously. In the microwell‐based dPCR reaction, the highly sensitive and specific target DNA quantification is achieved by applying polychromatic fluorescence‐labeled oligonucleotide probes. A specialized two‐dimensional image acquisition device would be adopted to acquire and analysis result images. During the process, the template in the well reacts specifically with the labeled probe to generate fluorescence signals, and each target molecule in the sample could be therefore identified and quantitatively analyzed.^49^

In addition to microwell‐based sample dispersion, sample dispersion approaches based on hydrogel beads were proposed. For example, research on the multiplexed hydrogel bead‐based dPCR have demonstrated that hydrogel microbeads and the emulsification technology performed excellently in producing micro‐droplets containing template chains.^50^, ^51^ In the first step of hydrogel bead‐based dPCR, common sequence marker primers are utilized to fabricate the reverse transcriptional template. Then, the single molecule amplification based on microemulsion is exerted. The beads on the hydrogel bead array were counted and the target concentration was finally determined. Furthermore, the detection of multiple targets could be realized by this hydrogel bead‐based dPCR strategy. Liu et al developed a DNA quantification strategy combining the emulsion PCR (emPCR) and the hydrogel bead‐array.^43^ As shown in Figure 2C, this assay consists of the following 3 steps: sDNA bisulfite conversion, PCR pre‐amplification, and emPCR to realize the single‐molecular amplification and the identification of hydrogel beads containing amplicons. By applying the hydrogel bead array‐based emPCR method, the highly sensitive and specific analysis of hypermethylated vimentin gene in clinical stool samples was successfully realized.

Besides the emerging dPCR strategies mentioned above, inkjet printing has shown significant advantages in the development of dPCR because of its unique flexibility and high‐throughput detection capability. Many studies have shown that the “printing‐based dPCR”, which combines drip printing and dPCR technique, was able to produce the customized instrument meets various requirements in different experiments.^52^, ^53^ There are usually three main steps in the droplet printing‐based dPCR. Firstly, the oil droplet array would be printed on the chemically modified hydrophilic‐in‐hydrophobic substrate with the hydrophilic pattern. Subsequently, PCR mixture as well as sample droplets containing target molecules are printed in each oil droplet, followed by the amplification cycle. At the end of the PCR reaction, fluorescent spot images were captured and quantitatively analyzed. Furthermore, research has shown that inspired by droplet printing, qPCR detection could also be carried out within hundreds of droplets which were printed on hydrophobic and oleophobic substrate.^54^, ^55^ The volume of the hydrophobic droplets encapsulated in silicone oil can be as low as 800 pL, thereby reducing the sample loss and the DNA contamination or cross‐contamination during thermal cycling.

## 3 NEXT GENERATION SEQUENCING‐BASED ctDNA DETECTION STRATEGIES

### 3.1 Targeted amplicon sequencing next generation sequencing systems

Next generation sequencing includes a series of methods that enable large‐scale sequence analysis of DNA or RNA. Next generation sequencing approaches allow for the analysis of heterogeneous samples and offer over 10 million randomly selected nucleic acid molecules sequence information. It is different from the first generation sequencing technology (Sanger sequencing), which is based on the DNA synthesis reactions and needs the same input DNA sample.^56^, ^57^ Due to its low sensitivity and the difficulty in short strand DNA sequencing, the traditional Sanger sequencing is less applied in ctDNA detection. In contrast, NGS techniques present with applicability for nucleic acid analysis and diagnosis that requires multiple analysis of many genes and their variations, since these strategies possess high detection throughput and satisfactory sensitivity. At present, two NGS‐based ctDNA detection strategies have been applied in the clinical trials. One of them is targeted amplicon sequencing (TAS); during the TAS process, the fragment where the single nucleotide polymorphism (SNP) site is located would be amplified by the PCR technique and uniformly added sequencing connectors and primers for the following NGS sequencing.^58^, ^59^, ^60^, ^61^, ^62^

### 3.2 Targeted capture sequencing next generation sequencing systems

**FIGURE 3:**
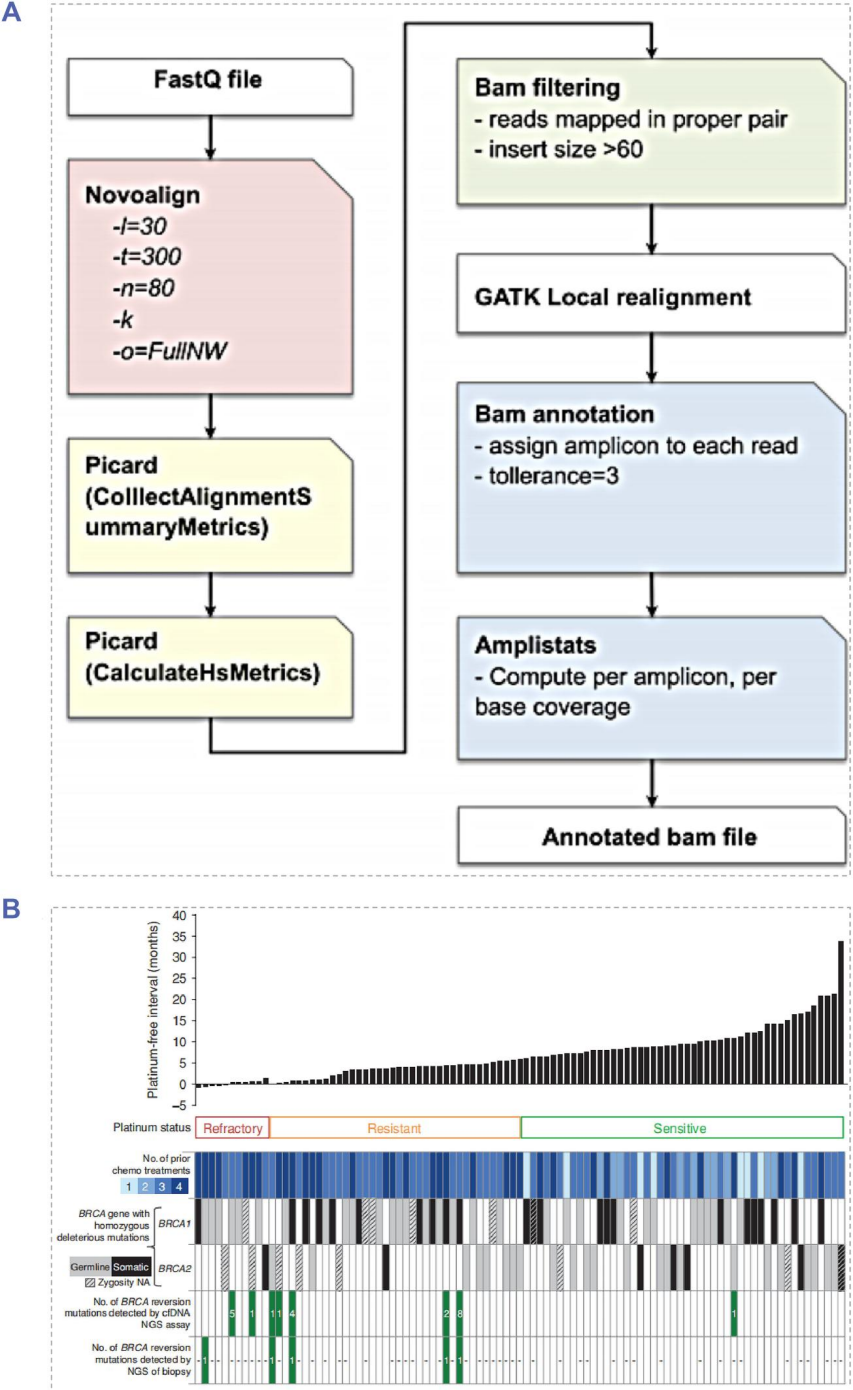
ctDNA analysis applying NGS‐based approaches. (A) Schematic illustration of the alignment and processing of the raw sequencing data. Reproduced under terms of the CC‐BY license.^61^ Copyright 2019, The Authors, published by Springer Nature. (B) Indication of the number of BRCA mutations in cfDNA and tumor biopsy of patients with HGOC and platinum resistance. Reproduced with permission.^65^ Copyright 2019, American Association for Cancer Research.

Different from the TAS method, in the process of the targeted capture sequencing (TCS), bio‐probes were utilized to retrieve the segment where SNP is located and then the NGS sequencing was conducted uniformly.^63^, ^64^ Gao et al. developed a TAS‐NGS system containing 377 amplicons of 20 cancer genes to detect ctDNA in metastatic breast cancer patients^61^ (Figure 3A). ctDNA mutations with an allele frequency greater than 1% in cfDNA was able to be monitored through this method. Lin et al. employed the TAS‐NGS method to investigate the relationship between BRCA mutated ctDNA in the blood sample of patients with ovarian carcinoma (HGOC) and platinum drug resistance^65^ (Figure 3B). The results indicated that there are BRCA mutations in the ctDNA of 18% (2/11) of clinical samples of platinum‐resistant HGOC patients and 13% (5/38) of clinical samples of platinum‐resistant HGOC patients. Although with many advantages and achievements, there are still non‐negligible problems restricting the wider application of NGS methods in ctDNA detection. All NGS systems may experience intrinsic error due to decreased enzyme specificity, signal ambiguity, imperfect deprotection, or other reasons. The calling of variants, especially of those rare variants in low, would become more difficult because of the sequencing errors. Recently, innovative research has been conducted on molecular barcodes, and the error rates in the NGS process were significantly reduced by increasing the sequencing depth.^66^, ^67^ There are currently no FDA‐approved NGS tests for tumor‐related diagnosis; however, the commercialized Foundation One LDT has been approved for marketing and has gained the attention from clinical oncologists.

## 4 EMERGING BIOSENSOR‐BASED ctDNA DETECTION STRATEGIES APPLIED IN CANCER THERANOSTICS

### 4.1 Electrochemical biosensors

Chip‐based Electrochemical biosensing systems are attractive alternatives for the analysis of clinical samples because of the cost‐effective instrument and equipment applied in the detection, resulting in the high feasibility to achieve the automation. Besides, they possess the high sensitivity and the adaptability to high‐level multiplexing, which meet the requirements of high‐throughput DNA or RNA analysis.^68^ By strictly controlling the detection conditions, previous research based on electrochemical technology have the achieved point‐mutation assay.^69^ Recent reports have indicated that electrochemical technology was successfully applied in analyzing various tumor markers and infectious pathogens in the clinical samples.^70^, ^71^ However, when mutated sequences in the clinical sample are mixed with a large number of wild‐type sequences, the detection selectivity shown using these methods would not be desirable enough. In comparison, the electrochemical biosensor‐based nucleic acid detection platform shows great application potential in field‐deployable analysis due to its high specificity, high sensitivity and rapid response.^72^ When the bio‐probe in the ctDNA electrochemical biosensor recognize the target ctDNA, this specific binding would be converted into detectable electrical signals. Electrochemistry technology‐based biosensors are able to obtain and analyze bioinformation from the single frequency or multifrequency band impedance.^73^, ^74^ Additionally, benefiting from the cost‐effectivity, portability, near‐realtime detection and other superiority, electrochemical biosensor‐based ctDNA assay system has become a desirable tool for the point of care testing and the mobile health monitoring.^24^, ^75^, ^76^

Electrochemical biosensor‐based ctDNA detection platforms mostly consist of three main components: the biomolecular recognition component, the conductive electrodes and the signal transduction system. Biomolecular recognition components could be composed of a variety of biological molecular receptors, including enzymes, immunoreactive substances, nucleic acids and organelles. These receptors are attached to the biosensor surface via affinity^77^, ^78^ covalent bonding,^79^, ^80^, ^81^ embedding,^82^, ^83^, ^84^ self‐assembling^85^, ^86^, ^87^ or other approaches and possess the capacity to precisely identify target analytes.^88^ DNA, antibodies, or other types of recognition elements could be stably fixed on the electrode through various surface modification techniques, including physical adsorption, while maintaining their biological activity, forming the conductive electrodes,^89^ which is the second part of the electrochemical biosensor‐based ctDNA detection system. Types of the commonly applied electrode materials include graphene, carbon and gold or other metals. Biochemistry reactions during the ctDNA detection could be transformed into detectable electrical signals and then further amplified or processed for the following quantitative analysis. In the signal transduction system, various electrochemical analysis methods including electrochemical impedance spectroscopy (EIS), cyclic voltammetric (CV), square‐wave voltammetry (SWV) and differential pulse voltammetry were utilized to determine the composition and content of the analyte according to the basic electrochemistry principles and the electrochemical properties of the analyte.

**FIGURE 4:**
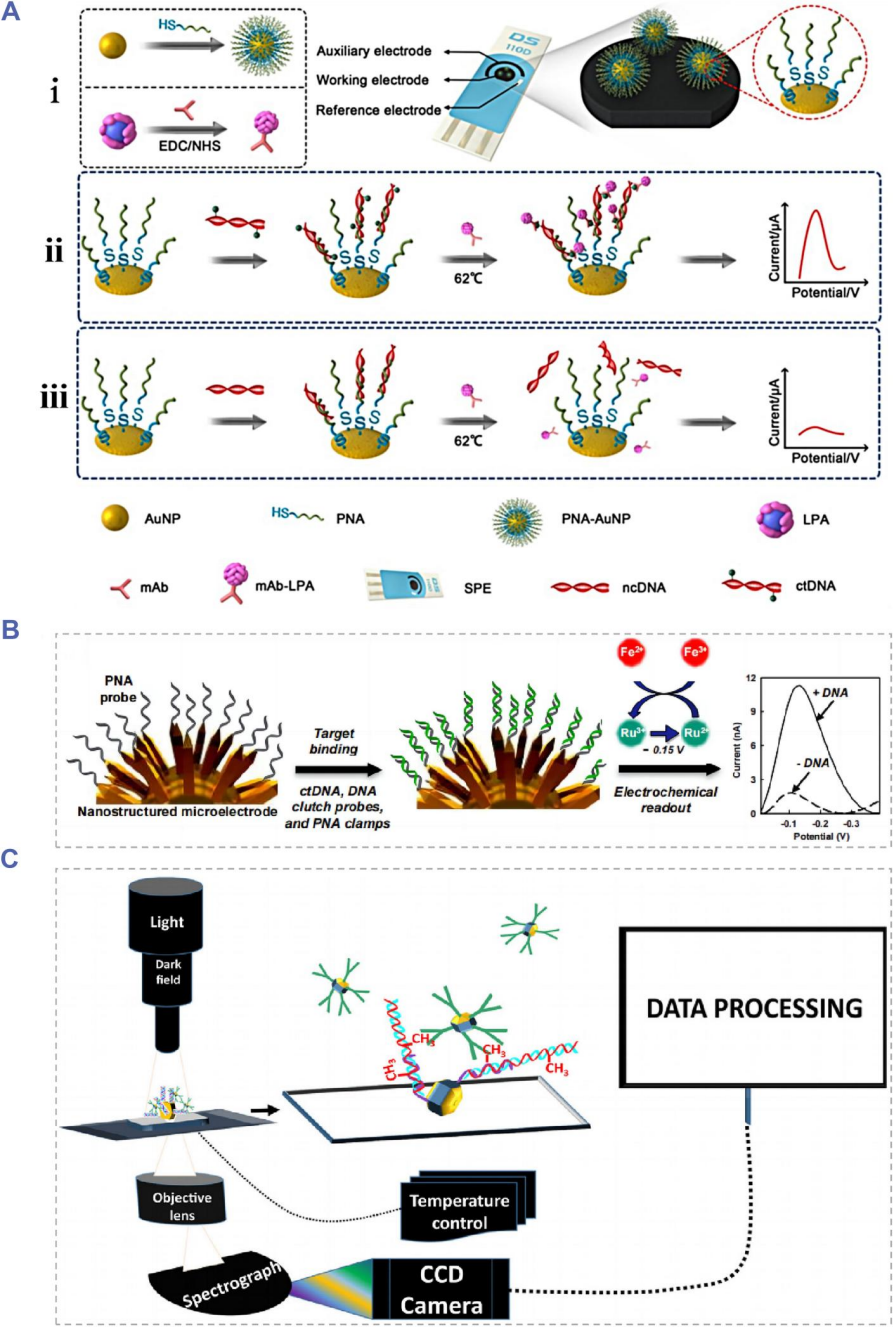
Electrochemical biosensors involved ctDNA detection platforms. (A) Schematic diagram of fabrication and the detection principle of the PNA‐AuNPs and LPA‐based DNA sensing platform. Reproduced with permission.^93^ Copyright 2018, The Authors, published by Ivyspring International Publisher. (B) Schematic illustration of the PNA‐probe‐based electrochemical biosensor for ctDNA analysis. Reproduced with permission.^94^ Copyright 2016, American Chemical Society. (C) Schematic of ctDNA and methylation detection system based on the gold nanoparticles and local surface plasmon resonance. Reproduced with permission.^95^ Copyright 2015, Elsevier.

Biosensor‐based nucleic acid analysis methods have been widely employed in biomedical fields such as clinical diagnosis and bacterium detection due to their high sensitivity and specificity, fast responsiveness, portability and ease of operation.^90^, ^91^, ^92^ Peptide nucleic acid (PNA), DNA, RNA and antibodies have already been applied as the recognition probes in electrochemical biosensor ctDNA detection systems. Based on PNA probes modified by the gold nanoparticle and the lead phosphate apolipoprotein, Cai et al. developed a dual biomarker assay platform^93^ (Figure 4A). This detection platform was used to quantify ctDNA, and to analyze the methylation and tumor characteristic mutations of the PIK3CA gene in clinical samples. Besides, as shown in Figure 4B, Das et al. presented a DNA clutch probe‐based biosensor which could prevent the ssDNA scaffold recombination. The selectivity was thus greatly improved compared with the previous PNA‐probe‐based ctDNA biosensor.^94^ Nguyen et al. realized the capture and enrichment of 69 bp PIK3CA ctDNA by applying a PNA probe.^95^ As exhibited in Figure 4C, via a coupled plasma model based on gold nanoparticles and local surface plasmon resonance (LSPR), the detection of the methylation of the PIK3CA gene and tumor characteristic mutations of the E542 K gene and E545 K gene was realized with the ultrahigh sensitivity.

**FIGURE 5:**
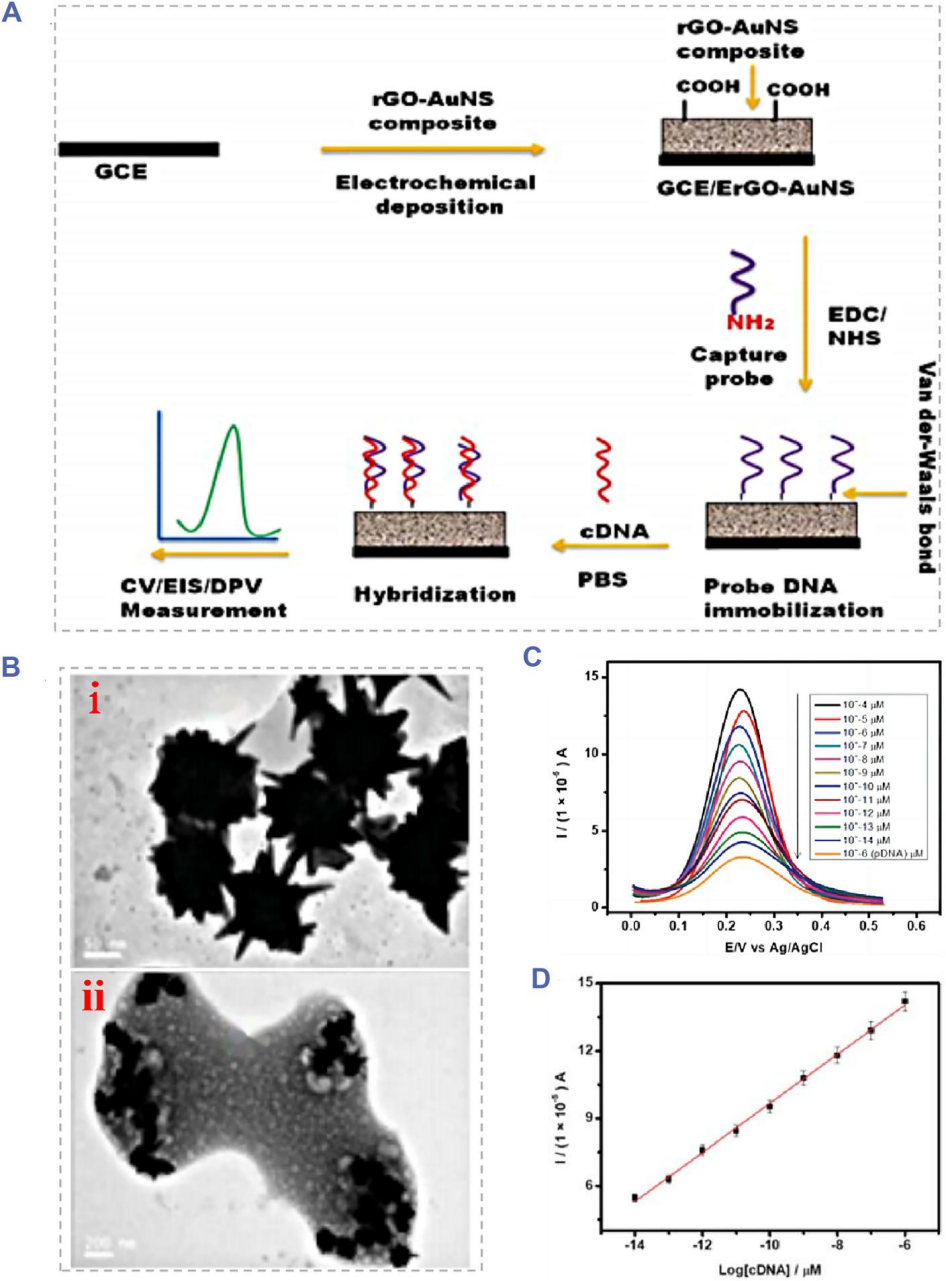
The graphene‐oxide‐decorated gold nanoparticles based electrochemical biosensing platform for ctDNA detection. (A) Schematic of the preparation process of the electrochemical biosensor. (B) TEM images of the (i) AuNSs and the (ii) rGO‐AuNS nanocomposite film. (C) The DPVs for ctDNA quantification. (D) The corresponding calibration curves for ctDNA detection of different concentrations. Reproduced with permission.^96^ Copyright 2020, The Royal Society of Chemistry.

**FIGURE 6:**
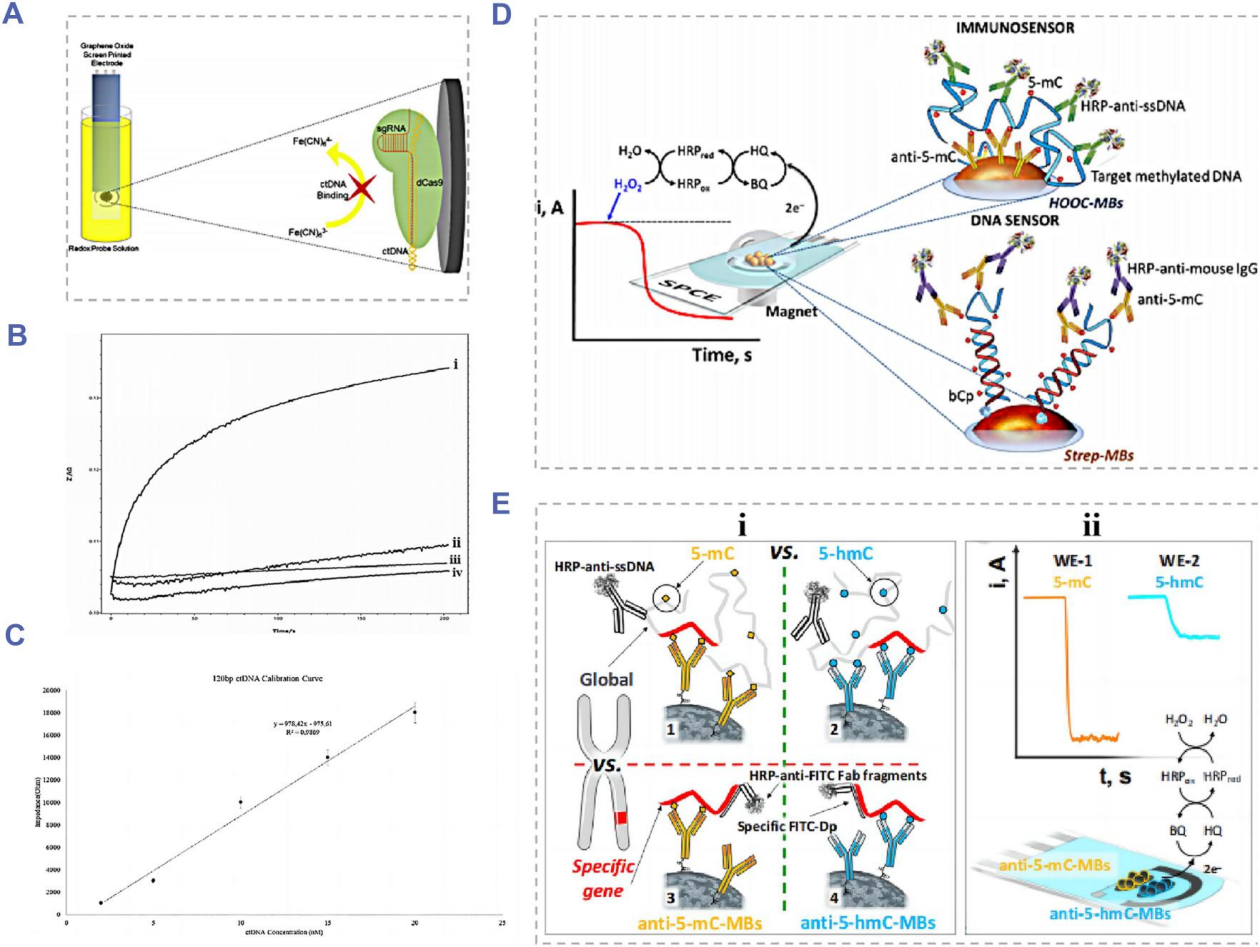
Examples of nucleic acid probe‐based or antibody‐based biosensors for ctDNA analysis. (A) Schematic illustration of the CRISPR‐dCas9‐sgRNA powered impedimetric biosensor for label‐free ctDNA detection. (B) Chronoimpedimetric results of (i) 120 bp mutant ctDNA, (ii) single nucleotide mutation ctDNA, (iii) 120 bp wild‐type ctDNA and (iv) non‐DNA included sample. (C) The calibration curves for ctDNA of 0, 2.5, 5, 10, 15 and 20 nM. Reproduced with permission.^100^ Copyright 2020, Elsevier. (D) Schematic display of the DNA sensor for the 5‐mC methylation detection applying the H_2_O_2_/HQ system at the screen‐printed carbon electrodes (SPCE). Reproduced under terms of the CC‐BY license.^101^ Copyright 2018, The Authors, published by Springer Nature. (E) Schematic design of the versatile electroanalytical biosensing system for the detection of cancer‐related DNA 5‐meth‐yl‐ and 5‐hydroxymethyl‐cytosines. Reproduced with permission.^102^ Copyright 2019, American Chemical Society.

For the real‐time ctDNA detection, Mahbubur et al. constructed a ctDNA detector by modifying the glassy carbon electrode with graphene‐oxide‐decorated gold nanoparticles and mounting the recognition probe via the π–π interaction between DNA bases.^96^ As displayed in Figure 5, by successfully detecting ctDNA of the PIK3CA gene in the peripheral blood of gastric cancer patients, this ctDNA analysis system showed great potential in the real‐time monitoring of ctDNA. Besides, with the uniform distribution of Au‐Pt alloy nanoparticles on MWCNT‐PDA, a novel nanocomposite ctDNA biosensor was successfully fabricated,^97^ which reduced the LoD (5 × 10^−16^ M) while improving the detection sensitivity. Furthermore, in order to realize more accurate biosensor‐based ctDNA detection, Chen et al exploited an electrochemistry biosensor using the gold platinum (AuPt)‐loaded 3‐dimensional‐graphene‐like homogeneous carbon structure (3D‐GHC600),^98^ which possessed the high satisfactory selectivity, reproducibility and excellent stability. By introducing the nanostructure transformation technique, Miao et al developed a highly specific DNA recognition system utilizing the principle of DNA bipedal walker and base complementary pairing of DNA nanostructure transformation.^99^ Uygun et al designed and fabricated a brand new biosensor‐based labeling free ctDNA detection platform, the biomolecular recognition component of this system was composed of an inactivated Cas9 (dCas9) protein and a silk‐screen printing graphene oxide electrode (GPHOXE) modifying using the specially designed synthetic guide RNA (sgRNA).^100^ As displayed in Figure 6A–C, the detection of tumor‐related mutations of PIK3CA exon could be achieved by the sequence‐specific recognition between the dCas9‐sgRNA‐modified biosensor and the target analyte and the assistance of EIS analysis. The dCas9‐sgRNA modified biosensing system is provided with rapid responsiveness and could realize ctDNA detection within 40 s with a linear range from 2 to 20 nM. The standard curve indicated the satisfactory linearity with the LoD determined to be 0.65 nM, and the limit of quantification determined to be 1.92 nM.

In contrast, the specific binding of antibodies to specific molecules is the basis for their application in the detection of molecules with biological characteristics, such as pathogens, cells, and bacteria. Antibody‐probe‐based detection methods have enormous application potential for their advantages of the decreased non‐specific interference and the lower LoD. Antibodies now have been widely applied in clinical diagnosis and therapy.^103^ As for these antibody‐involved ctDNA electrochemical biosensors, the target‐ctDNA‐specific antibody is fixed on the electrode to form the recognition component. During the detection process, the target ctDNA was captured and analyzed by electrochemistry or enzyme linked immunosorbent assay (ELISA). By applying the 5‐mC single antibody as the ctDNA‐specific receptor, Povedano et al developed the DNA analysis platform for detecting 5‐mC methylation on the basis of the screen‐printed carbon electrodes processed by a hydrogen peroxide/hydroquinone (H_2_O_2_/HQ) system^101^ (Figure 6D). Furthermore, Povedano et al proposed an antibody‐based electrochemical ctDNA immunosensor,^102^ which utilized two different antibodies and enabled detection of RNA methylation with an LoD of 1.25 × 10^−15^ M (Figure 6E). Compared with existing methods, the modified electrode of this immunosensor could physically adsorb the targets and specifically identify the ctDNA methylation, thus making the strategy simple, practical and cost‐effective. Therefore, these immunosensor systems exhibited great potential for wider applications in the on‐site detection and the mobile healthcare monitoring.

### 4.2 Surface plasmon resonance and Raman spectroscopy

**FIGURE 7:**
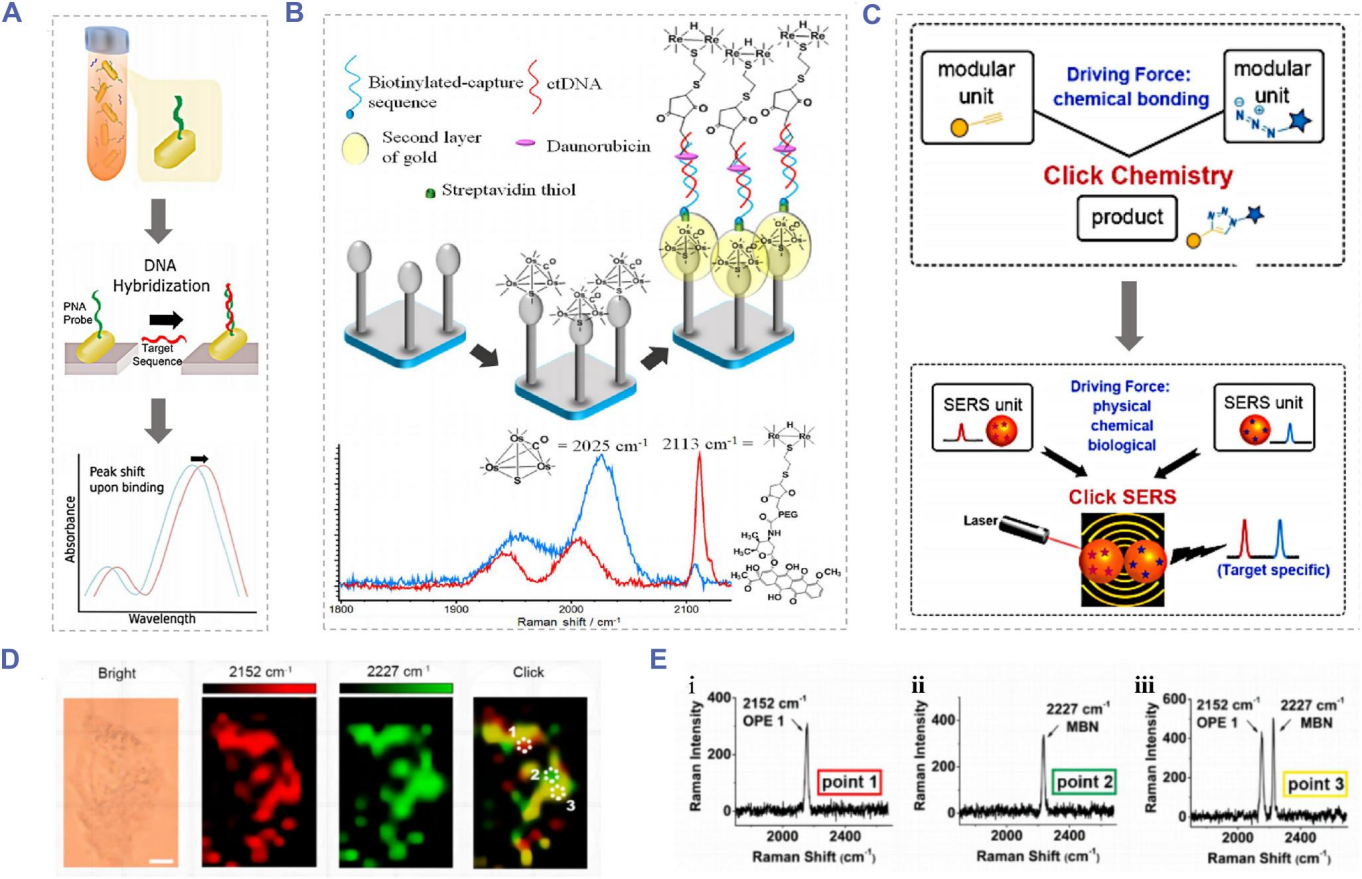
Surface plasmon resonance or LSPR‐based biosensors for ctDNA detection. (A) Schematic of the Peptide nucleic acid (PNA) probes‐modified gold nanorods for ctDNA point mutation detection. Reproduced with permission.^105^ Copyright 2019, Elsevier. (B) Schematic of the composition of Au−Os−CO−Au functionalized SERS‐active biosensor. Reproduced with permission.^106^ Copyright 2018, American Chemical Society. (C) Schematic demonstration of the principle of the“click surface‐enhanced Raman scattering (SERS)”detection strategy. (D) The SERS imaging of HeLa cells incubated with the Click SERS probes. (E) The SERS spectra of (i) point 1 with OPE1, (ii) point 2 with MBN and (iii) point 3 with two codes. The scale bar is 5 μm. Reproduced with permission.^107^ Copyright 2018, American Chemical Society.

Surface plasmon resonance or localized SPR (LSPR) possesses high sensitivity to refractivity changes near the sensing region generated by the interaction between specific macromolecules. Over recent years, they have attracted great interest of many researchers in related fields as an alternative promising nucleic acid analysis technology.^104^ Tadimety et al designed a gold nanorod‐based ctDNA analysis platform to detect the ctDNA point mutations with no need of the fluorescent labeling or amplifying.^105^ As displayed in Figure 7A, during the detection, PNA probes fixing on gold nanorods were capable of specifically recognizing the G12 V mutation in the KRAS gene, and the LSPR absorbance of the sample would be obtained after the ctDNA exposure. This method could be applied not only to distinguish the synthetic mutated and wild‐type DNA sequences but also to identify the mutated and the wild‐type DNA sequences of the KRAS gene in clinical samples. However, research on SPR‐based ctDNA detection strategies is relatively rare. The proximity needed for the effective plasma coupling of nanoparticles could be impeded by the ctDNA attachment, thus longer ctDNA fragments tend to inhibit the solution‐based aggregation. The typical ctDNA fragment size presented in clinical samples is about 150 bp, which may result in the limited utilization of these SPR‐based approaches. Besides, the formation of DNA secondary structures which could conceal mutated sequences may also be detrimental to the widespread application of this technology.

Due to its unsatisfactory sensitivity, there are few reports about the Raman spectroscopy technique applied in ctDNA detection. In contrast, surface‐enhanced Raman scattering (SERS) technology relies on the signal enhancement generated by the target molecule adsorbed on the surface of a metal conductor to realize quantitative analysis. The method has overcome this obstacle and had the advantages of high sensitivity, low cost and no enzyme involvement, showed significant potential for clinical ctDNA analysis.^108^ Zhou et al developed a DNA‐mediated SERS of single‐walled carbon nanotubes (SWNTs) and utilized the technique to detect ctDNA in human blood samples.^109^ Lin et al designed a SERS ratiometric assay system which was composed of a rhenium carbonyl (Re−CO) DNA probe, a SERS‐active substrate encapsulating with the osmium carbonyl (Os−CO) internal reference and a streptavidin layer fixing on the substrate surface^106^ (Figure 7B). In this assay process, the specific capture of target cfDNA was achieved by the hybridization of cfDNA with immobilized biotinylated probes. Thanks to the unique spectral characteristics of metal carbonyl compounds, this SERS ratiometric cfDNA detection method has been employed as an assay tool for the cfDNA from Epstein‐Barr virus and successfully realized the first‐time quantitation of cfDNA level in clinical blood samples from nasopharyngeal carcinoma patients. Furthermore, Zeng et al proposed an innovative readout strategy named “click” SERS for NDA detection.^107^ As shown in Figure 7C, relying on the small molecule reaction similar to those triggered by click chemistry, two SERS tags would dimerize when recognizing the target; then, the Raman scattering from the tagged dimer would result in an output, which represents the combinatory signal of individual nanoparticle.

### 4.3 Fluorescent biosensors

**FIGURE 8:**
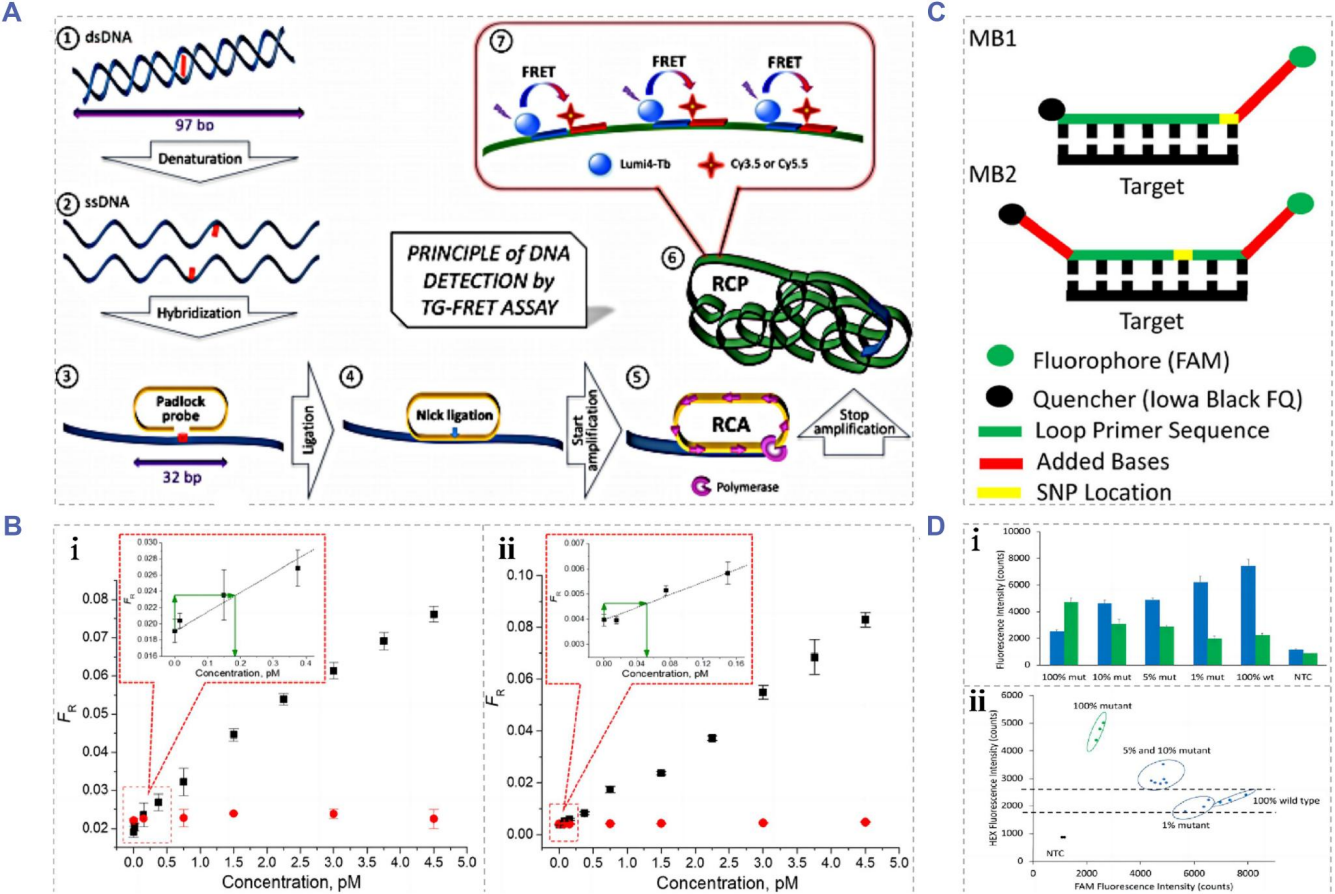
Fluorescent biosensor‐based ctDNA analysis platforms. (A) Schematic of the detection principle of the TG‐RCA‐FRET biosensor. (B) Calibration curves for (i) wild‐type dsDNA and (ii) mutated dsDNA in the concentration range from 0.015 to 4.5 p.m. Reproduced with permission.^110^ Copyright 2019, American Chemical Society. (C) Schematic illustrations of the molecular beacon probes applied in the ratiometric fluorescence assay system to detect mutated BRAF V600 E sequences. (D) The (i) fluorescence intensity value obtained employing the plate reader from reactions containing BRAF fragment with different concentrations of the mutated sequence, and the (ii) fluorescence plot demonstrating the mutant positive reactions. Reproduced with permission.^115^ Copyright 2020, American Chemical Society.

Forster resonance energy transfer (FRET) and the ratiometric fluorescence have the characteristics of high specificity, high accuracy and simple operation. Therefore, fluorescence biosensing systems based on those techniques may become promising candidates for novel cancer diagnostic strategies.^110^, ^111^, ^112^, ^113^, ^114^ Dekaliuk et al. proposed a FRET‐based ctDNA biosensing system, which integrated the isothermal rolling circle amplification and the gated FRET (TG‐FRET) of toluidine blue donors and two dye receptors (Cy3.5 and Cy5.5).^110^ As exhibited in Figure 8A,B, The TG‐RCA‐FRET biosensor posses satisfactory detection specificity of dsDNA with different chain lengths, and could detect the mutated with the Allele frequency of 1%. Quantitative analysis of wild‐type and mutated V600 E in the BRAF gene with a detection range of 75 fM to 45 p.m. was achieved by applying the TG‐RCA‐FRET biosensor, and with no need for washing and separation process. Although FRET‐based biosensing systems have many superiorities, their lower sensitivity compared to qPCR and dPCR restricts their wider application in ctDNA detection. In contrast, more accurate detection signals could be obtained through the ratio sensing approaches due to their self‐reference ability. On the basis of the molecular beacon (MB) probes and the loop‐mediated isothermal amplification (LAMP), Varona et al exploited a ratiometric fluorescence biosensing system for BRAFV600 E SNP analysis.^115^ As shown in Figure 8C,D, benefiting by the high specificity of MB probes and the rapid response and simple operation of LAMP, using a microplate reader as the detection device, the MB‐LAMP ratiometric fluorescence method could successfully detect mutated BRAFV600 E with 5% Allele frequency in the DNA mixture. The practical performance was significantly better than the traditional Sanger sequencing method (15%–20% Allele frequency). Further, with the assistance of solid‐phase microextraction, the detection of BRAFV600 E mutated ctDNA in human plasma samples was successfully achieved using the MB‐LAMP ratiometric fluorescence method, with the LoD as low as 73.26 fM.

## 5 CONCLUSION

ctDNA, as a liquid biopsy sample which could be obtained from peripheral blood through low invasive methods, is able to provide significant information such as tumor mutations spectrum, thus posses great potential in replacing the painful tissue biopsies and the application of tumor diagnosis, prognosis and treatment response monitoring. Herein, we present an overview of recent advances in ctDNA analysis systems, including typical dPCR‐based ctDNA detection methods, NGS‐based ctDNA assays, and emerging biosensor‐based ctDNA detection strategies. Through years of development, PCR‐based ctDNA analysis technologies have attained great progress. They evolved from the PCR target enrichment strategy to dPCR systems based on various sample dispersion methods including droplet sample dispersion, microwell sample dispersion and hydrogel bead‐based sample dispersion. These technical achievements have greatly reduced the LoD of PCR analysis systems and enabled them to break the limitation of detecting rare variations. However, during the PCR process, the known potential mutations of interest are necessary, and false negatives and false positive results caused by the contaminated chemicals in a clinical sample are also difficult to be completely avoided. NGS‐based strategy offers a method to detect mutations in a broader target space, while the high cost and long turnaround time still impede their wider application. Therefore, biosensor‐based ctDNA sensing platforms have become promising alternatives for clinical ctDNA analysis due to their high sensitivity, satisfactory feasibility and cost‐efficiency. ctDNA detection systems based on various biosensors (electrochemical biosensors, SPR or LSPR biosensors and fluorescent biosensors) have received widespread attention owing to their desirable nucleic acid analysis ability, potential commercial value and the feasibility to be integrated with a variety of analysis approaches. The research on these novel ctDNA analysis tools and liquid biopsy has made many significant advances with the potential of great biomedical impact, and is still continuously developing. In order to offer more innovative practicable technologies for clinical cancer therapy, biosensor‐based ctDNA detection systems still require further efforts to improve specificity and reproducibility. Besides, sufficient validation studies involving clinical samples are needed to demonstrate the feasibility and accuracy.

## AUTHOR CONTRIBUTIONS

Hongbo Zhang conceived the topic of the manuscript; Kexin Yi, Xiaoju Wang, Sergey K. Filippov, and Hongbo Zhang wrote the manuscript.

## CONFLICT OF INTEREST STATEMENT

Hongbo Zhang is an executive editor, and Sergey K. Filippov an associate editor of *Smart Medicine*. They were not involved in the editorial review or the decision to publish this article. All authors declare that there are no competing interests.

## ETHICS STATEMENT

Not applicable.

## References

[1] X. Ma , M. Mao , J. He , C. Liang , H. Xie , Chem. Soc. Rev. 2023, 52, 6447. 37615588 10.1039/d3cs00063j

[2] X. Wang , M. Zhang , Y. Li , H. Cong , B. Yu , Y. Shen , Small 2023, 2304006. 10.1002/smll.202304006 37635114

[3] C. Hassan , M. Spadaccini , Y. Mori , F. Foroutan , A. Facciorusso , P. Gkolfakis , G. Tziatzios , K. Triantafyllou , G. Antonelli , K. Khalaf , T. Rizkala , P. O. Vandvik , A. Fugazza , E. Rondonotti , J. R. Glissen‐Brown , S. Kamba , M. Maida , L. Correale , P. Bhandari , R. Jover , P. Sharma , D. K. Rex , A. Repici , Ann. Intern. Med. 2023, 176, 1209. 37639719 10.7326/M22-3678

[4] Q. Wang , Y. Zhi , M. Zi , Y. Mo , Y. Wang , Q. Liao , S. Zhang , Z. Gong , F. Wang , Z. Zeng , C. Guo , W. Xiong , Adv. Sci. 2023, 2302558. 10.1002/advs.202302558 PMC10602551 37632718

[5] J. H. Driskill , D. Pan , Nat. Rev. Mol. Cell Biol. 2023. 10.1038/s41580-023-00644-5 37626124

[6] J. Shen , N. Sun , J. Wang , P. Zens , T. Kunzke , A. Buck , V. M. Prade , Q. Wang , A. Feuchtinger , R. Hu , S. Berezowska , A. Walch , ACS Nano 2023, 17, 16396. 37639684 10.1021/acsnano.2c11161 PMC10510585

[7] X. Zhong , J. Luan , A. Yu , A. Lee‐Hassett , Y. Miao , L. Yang , Nucleic Acids Res. 2023, 51, e96. 37638762 10.1093/nar/gkad705 PMC10570049

[8] C. Zhai , J. Long , J. He , Y. Zheng , B. Wang , J. Xu , Y. Yang , L. Jiang , H. Yu , X. Ding , ACS Nano 2023, 17, 16656. 37638659 10.1021/acsnano.3c02853

[9] M. Poudineh , E. H. Sargent , K. Pantel , S. O. Kelley , Nat. Biomed. Eng. 2018, 2, 72. 31015625 10.1038/s41551-018-0190-5

[10] Y. van der Pol , F. Mouliere , Cancer Cell 2019, 36, 350. 31614115 10.1016/j.ccell.2019.09.003

[11] X. Yang , Z. Zhang , Y. Wu , H. Wang , Y. Yun , Y. Sun , H. Xie , B. Bogdanov , P. Senyushkin , J. Chi , Z. Lian , D. Wu , M. Su , Y. Song , Adv. Mater. 2023, 2304935. 10.1002/adma.202304935 37589665

[12] Y. Ma , J. Zhang , Y. Tian , Y. Fu , S. Tian , Q. Li , J. Yang , L. Zhang , Nat. Commun. 2023, 14, 4958. 37587113 10.1038/s41467-023-40668-1 PMC10432405

[13] K. K. Jakobsen , S. K. Bendtsen , N. Pallisgaard , J. Friborg , G. Lelkaitis , C. Grønhøj , C. von Buchwald , Clin. Cancer Res. 2023, 29, 3914. 37477909 10.1158/1078-0432.CCR-23-1064

[14] D. J. Carr , H. G. Welch , JAMA Intern. Med. 2023, 183, 1144. 37639262 10.1001/jamainternmed.2023.3603

[15] G. Ishigane , K. Toda , M. Tamamitsu , H. Shimada , V. R. Badarla , T. Ideguchi , Light Sci. Appl. 2023, 12, 174. 37463888 10.1038/s41377-023-01214-2 PMC10354118

[16] L. Wang , Y. Zhuang , Y. Yu , Z. Guo , Q. Guo , L. Qiao , X. Wang , X. Liang , P. Zhang , Q. Li , C. Huang , R. Cong , Y. Li , B. Che , H. Xiong , G. Lin , M. Rao , R. Hu , W. Wang , G. Yang , J. Lou , Biosens. Bioelectron. 2023, 238, 115548. 37542979 10.1016/j.bios.2023.115548

[17] H. A. Parsons , T. Blewett , X. Chu , S. Sridhar , K. Santos , K. Xiong , V. G. Abramson , A. Patel , J. Cheng , A. Brufsky , J. Rhoades , J. Force , R. Liu , T. A. Traina , L. A. Carey , M. F. Rimawi , K. D. Miller , V. Stearns , J. Specht , C. Falkson , H. J. Burstein , A. C. Wolff , E. P. Winer , N. Tayob , I. E. Krop , G. M. Makrigiorgos , T. R. Golub , E. L. Mayer , V. A. Adalsteinsson , Ann. Oncol. 2023, 34, 899. 37597579 10.1016/j.annonc.2023.08.004 PMC10898256

[18] J. C. Yang , G. Liu , S. Lu , J. He , M. Burotto , M. J. Ahn , D. W. Kim , X. Liu , Y. Zhao , S. Vincent , J. Yin , X. Ma , H. M. Lin , S. Popat , J. Thorac. Oncol. 2023. 10.1016/j.jtho.2023.08.010 37574132

[19] T. Powles , Z. J. Assaf , V. Degaonkar , P. Grivas , M. Hussain , S. Oudard , J. E. Gschwend , P. Albers , D. Castellano , H. Nishiyama , S. Daneshmand , S. Sharma , H. Sethi , A. Aleshin , Y. Shi , N. Davarpanah , C. Carter , J. Bellmunt , S. Mariathasan , Eur. Urol. 2023. 10.1016/j.eururo.2023.06.007 37500339

[20] C. Herberts , A. W. Wyatt , Trends Cancer 2021, 7, 995. 34219051 10.1016/j.trecan.2021.06.001

[21] S. H. Lee , S. M. Park , B. N. Kim , O. S. Kwon , W. Y. Rho , B. H. Jun , Biosens. Bioelectron. 2019, 141, 111448. 31252258 10.1016/j.bios.2019.111448

[22] Z. Diao , D. Han , R. Zhang , J. Li , J. Adv. Res. 2022, 38, 201. 35572406 10.1016/j.jare.2021.09.012 PMC9091713

[23] A. R. Ferhan , J. A. Jackman , J. H. Park , N. J. Cho , D. H. Kim , Adv. Drug Delivery Rev. 2018, 125, 48. 10.1016/j.addr.2017.12.004 29247763

[24] X. Li , M. Ye , W. Zhang , D. Tan , N. Jaffrezic‐Renault , X. Yang , Z. Guo , Biosens. Bioelectron. 2019, 126, 596. 30502682 10.1016/j.bios.2018.11.037

[25] N. R. Beer , B. J. Hindson , E. K. Wheeler , S. B. Hall , K. A. Rose , I. M. Kennedy , B. W. Colston , Anal. Chem. 2007, 79, 8471. 17929880 10.1021/ac701809w

[26] W. Guan , L. Chen , T. D. Rane , T. H. Wang , Sci. Rep. 2015, 5, 13795. 26333806 10.1038/srep13795 PMC4558716

[27] B. J. Hindson , K. D. Ness , D. A. Masquelier , P. Belgrader , N. J. Heredia , A. J. Makarewicz , I. J. Bright , M. Y. Lucero , A. L. Hiddessen , T. C. Legler , T. K. Kitano , M. R. Hodel , J. F. Petersen , P. W. Wyatt , E. R. Steenblock , P. H. Shah , L. J. Bousse , C. B. Troup , J. C. Mellen , D. K. Wittmann , N. G. Erndt , T. H. Cauley , R. T. Koehler , A. P. So , S. Dube , K. A. Rose , L. Montesclaros , S. Wang , D. P. Stumbo , S. P. Hodges , S. Romine , F. P. Milanovich , H. E. White , J. F. Regan , G. A. Karlin‐Neumann , C. M. Hindson , S. Saxonov , B. W. Colston , Anal. Chem. 2011, 83, 8604. 22035192 10.1021/ac202028g PMC3216358

[28] L. Mazutis , J. C. Baret , P. Treacy , Y. Skhiri , A. F. Araghi , M. Ryckelynck , V. Taly , A. D. Griffiths , Lab Chip 2009, 9, 2902. 19789742 10.1039/b907753g

[29] M. C. Strain , S. M. Lada , T. Luong , S. E. Rought , S. Gianella , V. H. Terry , C. A. Spina , C. H. Woelk , D. D. Richman , PLoS One 2013, 8, e55943. 23573183 10.1371/journal.pone.0055943 PMC3616050

[30] A. Tay , R. P. Kulkarni , A. Karimi , D. Di Carlo , Lab Chip 2015, 15, 4379. 26486454 10.1039/c5lc90117k

[31] P. Wang , F. Jing , G. Li , Z. Wu , Z. Cheng , J. Zhang , H. Zhang , C. Jia , Q. Jin , H. Mao , J. Zhao , Biosens. Bioelectron. 2015, 74, 836. 26232679 10.1016/j.bios.2015.07.048

[32] R. Dangla , S. C. Kayi , C. N. Baroud , Proc. Natl. Acad. Sci. U. S. A. 2013, 110, 853. 23284169 10.1073/pnas.1209186110 PMC3549071

[33] C. Zhang , D. Xing , Chem. Rev. 2010, 110, 4910. 20394378 10.1021/cr900081z

[34] Y. Chu , B. Cai , Y. Ma , Mi. Zhao , Z. Ye , J. Huang , RSC Adv. 2016, 6, 22673.

[35] D. J. Eastburn , A. Sciambi , A. R. Abate , Anal. Chem. 2013, 85, 8016. 23885761 10.1021/ac402057q

[36] T. Schneider , J. Kreutz , D. T. Chiu , Anal. Chem. 2013, 85, 3476. 23495853 10.1021/ac400257c PMC3631535

[37] V. Taly , D. Pekin , L. Benhaim , S. K. Kotsopoulos , D. Le Corre , X. Li , I. Atochin , D. R. Link , A. D. Griffiths , K. Pallier , H. Blons , O. Bouche , B. Landi , J. B. Hutchison , P. Laurent‐Puig , Clin. Chem. 2013, 59, 1722. 23938455 10.1373/clinchem.2013.206359

[38] C. Bettegowda , M. Sausen , R. J. Leary , I. Kinde , Y. Wang , N. Agrawal , B. R. Bartlett , H. Wang , B. Luber , R. M. Alani , E. S. Antonarakis , N. S. Azad , A. Bardelli , H. Brem , J. L. Cameron , C. C. Lee , L. A. Fecher , G. L. Gallia , P. Gibbs , D. Le , R. L. Giuntoli , M. Goggins , M. D. Hogarty , M. Holdhoff , S. M. Hong , Y. Jiao , H. H. Juhl , J. J. Kim , G. Siravegna , D. A. Laheru , C. Lauricella , M. Lim , E. J. Lipson , S. K. Marie , G. J. Netto , K. S. Oliner , A. Olivi , L. Olsson , G. J. Riggins , A. Sartore‐Bianchi , K. Schmidt , M. Shih l , S. M. Oba‐Shinjo , S. Siena , D. Theodorescu , J. Tie , T. T. Harkins , S. Veronese , T. Wang , J. D. Weingart , C. L. Wolfgang , L. D. Wood , D. Xing , R. H. Hruban , J. Wu , P. J. Allen , C. M. Schmidt , M. A. Choti , V. E. Velculescu , K. W. Kinzler , B. Vogelstein , N. Papadopoulos , L. A. Diaz Jr. , Sci. Transl. Med. 2014, 6, 224ra24. 10.1126/scitranslmed.3007094 PMC4017867 24553385

[39] G. R. Oxnard , C. P. Paweletz , Y. Kuang , S. L. Mach , A. O’Connell , M. M. Messineo , J. J. Luke , M. Butaney , P. Kirschmeier , D. M. Jackman , P. A. Janne , Clin. Cancer Res. 2014, 20, 1698. 24429876 10.1158/1078-0432.CCR-13-2482 PMC3959249

[40] S. J. Dawson , D. W. Tsui , M. Murtaza , H. Biggs , O. M. Rueda , S. F. Chin , M. J. Dunning , D. Gale , T. Forshew , B. Mahler‐Araujo , S. Rajan , S. Humphray , J. Becq , D. Halsall , M. Wallis , D. Bentley , C. Caldas , N. Rosenfeld , N. Engl. J. Med. 2013, 368, 1199. 23484797 10.1056/NEJMoa1213261

[41] H. Gevensleben , I. Garcia‐Murillas , M. K. Graeser , G. Schiavon , P. Osin , M. Parton , I. E. Smith , A. Ashworth , N. C. Turner , Clin. Cancer Res. 2013, 19, 3276. 23637122 10.1158/1078-0432.CCR-12-3768 PMC6485473

[42] S. C. Tsao , J. Weiss , C. Hudson , C. Christophi , J. Cebon , A. Behren , A. Dobrovic , Sci. Rep. 2015, 5, 11198. 26095797 10.1038/srep11198 PMC4476039

[43] Y. Liu , H. Wu , Q. Zhou , Q. Song , J. Rui , B. Zou , G. Zhou , Biosens. Bioelectron. 2017, 92, 596. 27829567 10.1016/j.bios.2016.10.054

[44] M. F. Sanmamed , S. Fernandez‐Landazuri , C. Rodriguez , R. Zarate , M. D. Lozano , L. Zubiri , J. L. Perez‐Gracia , S. Martin‐Algarra , A. Gonzalez , Clin. Chem. 2015, 61, 297. 25411185 10.1373/clinchem.2014.230235

[45] A. L. Reid , J. B. Freeman , M. Millward , M. Ziman , E. S. Gray , Clin. Biochem. 2015, 48, 999. 25523300 10.1016/j.clinbiochem.2014.12.007

[46] M. Versluis , M. J. de Lange , S. I. van Pelt , C. A. Ruivenkamp , W. G. Kroes , J. Cao , M. J. Jager , G. P. Luyten , P. A. van der Velden , PLoS One 2015, 10, e0116371. 25764247 10.1371/journal.pone.0116371 PMC4357379

[47] S. Lindstrom , H. Andersson‐Svahn , Biochim. Biophys. Acta 2011, 1810, 308. 20451582 10.1016/j.bbagen.2010.04.009

[48] C. Brenan , T. Morrison , Drug Discovery Today: Technol. 2005, 2, 247. 10.1016/j.ddtec.2005.08.017 24981943

[49] R. L. Stears , T. Martinsky , M. Schena , Nat. Med. 2003, 9, 140. 12514728 10.1038/nm0103-140

[50] Z. Qi , Y. Ma , L. Deng , H. Wu , G. Zhou , T. Kajiyama , H. Kambara , Analyst 2011, 136, 2252. 21509397 10.1039/c0an00976h

[51] R. Williams , S. G. Peisajovich , O. J. Miller , S. Magdassi , D. S. Tawfik , A. D. Griffiths , Nat. Methods 2006, 3, 545. 16791213 10.1038/nmeth896

[52] J. Hoffmann , S. Hin , F. von Stetten , R. Zengerle , G. Roth , RSC Adv. 2012, 2, 3885.

[53] S. Sakakihara , S. Araki , R. Iino , H. Noji , Lab Chip 2010, 10, 3355. 21031171 10.1039/c0lc00062k

[54] Y. Sun , X. Zhou , Y. Yu , Lab Chip 2014, 14, 3603. 25070461 10.1039/c4lc00598h

[55] Y. Zhu , Y. Zhang , W. Liu , Y. Ma , Q. Fang , B. Yao , Sci. Rep. 2015, 5, 9551. 25828383 10.1038/srep09551 PMC4381353

[56] E. L. Van Dijk , H. Auger , Y. Jaszczyszyn , C. Thermes , Trends Genet. 2014, 30, 418. 25108476 10.1016/j.tig.2014.07.001

[57] M. Eisenstein , Nat. Biotechnol. 2012, 30, 1023. 23138289 10.1038/nbt.2412

[58] D. S. Guttery , K. Page , A. Hills , L. Woodley , S. D. Marchese , B. Rghebi , R. K. Hastings , J. Luo , J. H. Pringle , J. Stebbing , R. C. Coombes , S. Ali , J. A. Shaw , Clin. Chem. 2015, 61, 974. 25979954 10.1373/clinchem.2015.238717

[59] K. Page , D. S. Guttery , D. Fernandez‐garcia , A. Hills , R. K. Hastings , J. Luo , K. Goddard , V. Shahin , L. Woodley‐barker , B. M. Rosales , R. C. Coombes , J. Stebbing , J. A. Shaw , Clin. Chem. 2017, 63, 532. 27940449 10.1373/clinchem.2016.261834 PMC6241835

[60] A. W. Hahn , D. M. Gill , B. Maughan , A. Agarwal , L. Arjyal , S. Gupta , J. Streeter , E. Bailey , S. K. Pal , N. Agarwal , Oncotarget 2017, 8, 33614. 28431395 10.18632/oncotarget.16833 PMC5464894

[61] M. Gao , M. Callari , E. Beddowes , S. J. Sammut , M. Grzelak , H. Biggs , L. Jones , A. Boumertit , S. C. Linn , J. Cortes , M. Oliveira , R. Baird , S. F. Chin , C. Caldas , Genome Med. 2019, 11, 1. 30609936 10.1186/s13073-018-0611-9 PMC6320579

[62] B. Wang , S. Wu , F. Huang , M. Shen , H. Jiang , Y. Yu , Q. Yu , Y. Yang , Y. Zhao , Y. Zhou , B. Pan , T. Liu , W. Guo , Clin. Chem. Lab. Med. 2019, 57, 1501. 31339850 10.1515/cclm-2019-0142

[63] Y. Wang , P. Tian , W. Wang , K. Wang , Z. Zhang , B. Chen , Y. He , L. Li , H. Liu , S. Chuai , W. Li , Oncotarget 2016, 7, 65208. 27564104 10.18632/oncotarget.11569 PMC5323149

[64] P. Sun , C. Chen , Y. Xia , Y. Wang , P. Liu , X. Bi , Y. Shao , Q. Ou , X. Wu , H. Yang , M. Nie , X. Zhang , Z. Li , W. Jiang , J. Cancer 2019, 10, 323. 30719126 10.7150/jca.27615 PMC6360295

[65] K. K. Lin , M. I. Harrell , A. M. Oza , A. Oaknin , I. Ray‐coquard , A. V. Tinker , E. Helman , M. R. Radke , C. Say , L. T. Vo , E. Mann , J. D. Isaacson , L. Maloney , D. M. O' Malley , S. K. Chambers , S. H. Kaufmann , C. L. Scott , G. E. Konecny , R. L. Coleman , J. X. Sun , H. Giordano , J. D. Brenton , T. C. Harding , I. A. Mcneish , E. M. Swisher , Cancer Discov. 2019, 9, 210. 30425037 10.1158/2159-8290.CD-18-0715

[66] R. Squillace , G. Frampton , P. J. Stephens , J. S. Ross , V. A. Miller , OncoTargets Ther. 2015, 8, 2237. 10.2147/OTT.S88908 PMC4556029 26346763

[67] E. Borgstrom , D. Redin , S. Lundin , E. Berglund , A. F. Andersson , A. Ahmadian , Nat. Commun. 2015, 6, 7173. 26055759 10.1038/ncomms8173 PMC4468844

[68] S. O. Kelley , C. A. Mirkin , D. R. Walt , R. F. Ismagilov , M. Toner , E. H. Sargent , Nat. Nanotechnol. 2014, 9, 969. 25466541 10.1038/nnano.2014.261 PMC4472305

[69] Y. Wu , R. Lai , Chem. Commun. 2013, 49, 3422. 10.1039/c3cc41281d 23503676

[70] J. D. Besant , J. Das , I. B. Burgess , W. Liu , E. H. Sargent , S. O. Kelley , Nat. Commun. 2015, 6, 6978. 25901450 10.1038/ncomms7978 PMC4421844

[71] J. Das , S. O. Kelley , Anal. Chem. 2013, 85, 7333. 23799266 10.1021/ac401221f

[72] M. Sharifi , A. Hasan , F. Attar , A. Taghizadeh , M. Falahati , Talanta 2020, 217, 121091. 32498898 10.1016/j.talanta.2020.121091

[73] F. Chen , X. Wang , X. Cao , Y. Zhao , Anal. Chem. 2017, 89, 10468. 28810735 10.1021/acs.analchem.7b02572

[74] R. Antiochia , Biosens. Bioelectron. 2021, 173, 112777. 33189015 10.1016/j.bios.2020.112777 PMC7591947

[75] N. M. Sumita , C. E. S. Ferreira , M. D. V. Martino , C. N. Franca , A. C. L. Faulhaber , M. Scartezini , J. R. R. Pinho , C. M. Dias , K. R. Cesar , V. M. Pariz , J. C. C. Guerra , I. V. Barbosa , M. H. W. Faulhaber , M. C. Batista , A. Andriolo , M. E. Mendes , A. M. O. Machado , M. P. Colombini , N. Slhessarenko , W. Shcolnik , C. Khawali , G. A. Campana , F. Berlitz , C. A. Galoro , Clin. Lab. 2018, 64, 1105. 30146832 10.7754/Clin.Lab.2018.171021

[76] J. Das , S. O. Kelley , Angew. Chem. Int. Ed. 2020, 59, 2554. 10.1002/anie.201905005 31332937

[77] H. Kim , M. Tran , E. Petryayeva , O. Solodova , K. Susumu , E. Oh , I. Medintz , W. Algar , ACS Appl. Mater. Interfaces 2020, 12, 53462. 33180467 10.1021/acsami.0c14559

[78] S. Bormann , B. Burek , R. Ulber , D. Holtmann , Mol. Catal. 2020, 492, 110999.

[79] B. Jiang , H. Yu , Y. Zhang , H. Feng , S. Hoag , Pharm. Res. 2017, 34, 2663. 28808837 10.1007/s11095-017-2237-9 PMC5738263

[80] C. Armbruster , D. Wolter , M. Mishra , H. Hayden , M. Radey , G. Merrihew , M. MacCoss , J. Burns , D. Wozniak , M. Parsek , L. R. Hoffman , mBio 2016, 7, e00538‐16. 27222468 10.1128/mBio.00538-16 PMC4895107

[81] B. Hiebl , L. Ascher , K. Luetzow , K. Kratz , C. Gruber , C. Mrowietz , M. Nehring , A. Lendlein , R. Franke , F. Jung , Clin. Hemorheol. Microcirc. 2018, 69, 317. 29630534 10.3233/CH-189108

[82] C. Stan , P. Horlescu , L. Ursu , M. Popa , C. Albu , J. Mater. Sci. 2017, 52, 185.

[83] J. Kulicek , P. Gemeiner , M. Omastová , M. Micusik , Chem. Pap. Slovak Acad. Sci. 2018, 72, 1651.

[84] A. Hassanein , N. Salahuddin , A. Matsuda , T. Hattori , M. Elfiky , Electroanalysis 2018, 30, 459.

[85] C. Cui , Y. Deng , L. Han , Sci. China Mater. 2020, 63, 686. 32219007 10.1007/s40843-019-1261-1 PMC7094945

[86] H. Luo , X. Lin , Z. Peng , M. Song , L. Jin , Micromachines 2020, 11, 41. 10.3390/mi11010041 PMC7019973 31905833

[87] K. Dey , S. Bhunia , H. S. Sasmal , C. M. Reddy , R. Banerjee , J. Am. Chem. Soc. 2021, 143, 955. 33406365 10.1021/jacs.0c11122

[88] J. Chambers , B. Arulanandam , L. Matta , A. Weis , J. Valdes , Curr. Issues Mol. Biol. 2008, 10, 1. 18525101

[89] H. A. Abdulbari , E. Basheer , ChemBioEng Rev. 2017, 4, 92.

[90] G. Yao , H. Pei , J. Li , Y. Zhao , D. Zhu , Y. Zhang , Y. Lin , Q. Huang , C. Fan , NPG Asia Mater. 2015, 7, e159.

[91] F. Yang , X. Zuo , Z. Li , W. Deng , J. Shi , G. Zhang , Q. Huang , S. Song , C. Fan , Adv. Mater. 2014, 26, 4671. 24729272 10.1002/adma.201400451

[92] Z. Ge , H. Pei , L. Wang , S. Song , C. Fan , Sci. China Chem. 2011, 54, 1273.

[93] C. Cai , Z. Guo , Y. Cao , W. Zhang , Y. Chen , Nanotheranostics 2018, 2, 12. 29291160 10.7150/ntno.22419 PMC5743835

[94] J. Das , I. Ivanov , E. Sargent , S. Kelley , J. Am. Chem. Soc. 2016, 138, 11009. 27513828 10.1021/jacs.6b05679

[95] A. H. Nguyen , S. J. Sim , Biosens. Bioelectron. 2015, 67, 443. 25220802 10.1016/j.bios.2014.09.003

[96] M. Rahman , D. Cui , S. Zhou , A. Zhang , D. Chen , Anal. Methods 2020, 12, 440.

[97] H. Zhao , Z. Niu , K. Chen , L. Chen , Z. Wang , M. Lan , J. Shi , W. Huang , Microchem. J. 2021, 171, 106783.

[98] K. Chen , H. Zhao , Z. Wang , M. Lan , Mater. Today Chem. 2022, 24, 100892.

[99] P. Miao , H. Chai , Y. Tang , ACS Nano 2022, 16, 4726. 35188755 10.1021/acsnano.1c11582

[100] Z. O. Uygun , L. Yeniay , F. G. Sağin , Anal. Chim. Acta 2020, 1121, 35. 32493587 10.1016/j.aca.2020.04.009

[101] E. Povedano , E. Vargas , V. Montiel , R. Torrente‐Rodriguez , M. Pedrero , R. Barderas , P. S. Segundo‐Acosta , A. Pelaez‐Garcia , M. Mendiola , D. Hardisson , S. Campuzano , J. M. Pingarrón , Sci. Rep. 2018, 8, 6418. 29686400 10.1038/s41598-018-24902-1 PMC5913137

[102] E. Povedano , V. Montiel , A. Valverde , F. Navarro‐Villoslada , P. Yanez‐Sedeno , M. Pedrero , A. Montero‐Calle , R. Barderas , A. Pelaez‐Garcia , M. Mendiola , D. Hardisson , J. Feliú , J. Camps , E. Rodríguez‐Tomàs , J. Joven , M. Arenas , S. Campuzano , J. M. Pingarrón , ACS Sens. 2019, 4, 227. 30499292 10.1021/acssensors.8b01339

[103] K. Kano , O. Shirai , Y. Kitazumi , K. Sakai , H. Xia , in Enzymatic Bioelectrocatalysis, Springer, Singapore 2021, pp. 105–114.

[104] A. R. Ferhan , J. A. Jackman , J. H. Park , N. J. Cho , D. H. Kim , Adv. Drug Delivery Rev. 2018, 125, 48. 10.1016/j.addr.2017.12.004 29247763

[105] A. Tadimety , Y. Zhang , K. M. Kready , T. J. Palinski , G. J. Tsongalis , J. X. J. Zhang , Biosens. Bioelectron. 2019, 130, 236. 30769288 10.1016/j.bios.2019.01.045

[106] D. Lin , T. Gong , Z. Y. Hong , S. Qiu , J. Pan , C. Y. Tseng , S. Feng , R. Chen , K. V. Kong , Anal. Chem. 2018, 90, 7139. 29808995 10.1021/acs.analchem.8b01931

[107] Y. Zeng , J. Q. Ren , A. G. Shen , J. M. Hu , J. Am. Chem. Soc. 2018, 140, 10649. 29975521 10.1021/jacs.8b04892

[108] Y. Zhang , X. Mi , X. Tan , R. Xiang , Theranostics 2019, 9, 491. 30809289 10.7150/thno.29875 PMC6376192

[109] Q. Zhou , J. Zheng , Z. Qing , M. Zheng , J. Yang , S. Yang , L. Ying , R. Yang , Anal. Chem. 2016, 88, 4759. 27028517 10.1021/acs.analchem.6b00108

[110] M. Dekaliuk , X. Qiu , F. Troalen , P. Busson , N. Hildebrandt , ACS Sens. 2019, 4, 2786. 31577130 10.1021/acssensors.9b01420

[111] M. Lu , X. Zhang , D. Xu , N. Li , Y. Zhao , Adv. Mater. 2023, 35, 2211330. 10.1002/adma.202211330 36905684

[112] X. Ren , L. Huang , C. Wang , Y. Ge , K. Zhang , D. Jiang , X. Liu , Q. Zhang , Y. Wang , Engineered Regen. 2022, 3, 387.

[113] T. Anazawa , H. Matsunaga , S. Yamamoto , R. Inaba , Lab Chip 2020, 20, 1083. 32108835 10.1039/c9lc00853e

[114] W. Zhou , L. Wang , C. Liu , Q. Teng , Z. Wang , Z. Dai , Chem. Sci. 2020, 11, 3745. 34094063 10.1039/c9sc06408g PMC8152624

[115] M. Varona , D. R. Eitzmann , D. Pagariya , R. K. Anand , J. L. Anderson , Anal. Chem. 2020, 92, 3346. 31950824 10.1021/acs.analchem.9b05323 PMC7155775

